# Utility of protein–protein binding surfaces composed of anti-parallel alpha-helices and beta-sheets selected by phage display

**DOI:** 10.1016/j.jbc.2024.107283

**Published:** 2024-04-11

**Authors:** Ningyu Zhu, Philip M. Smallwood, Amir Rattner, Tao-Hsin Chang, John Williams, Yanshu Wang, Jeremy Nathans

**Affiliations:** 1 Department of Molecular Biology and Genetics, Johns Hopkins University School of Medicine, Baltimore, USA; 2 Howard Hughes Medical Institute, Johns Hopkins University School of Medicine, Baltimore, USA; 3 Department of Neuroscience, Johns Hopkins University School of Medicine, Baltimore, USA; 4 Department of Ophthalmology, Johns Hopkins University School of Medicine, Baltimore, USA

**Keywords:** phage display, protein engineering, protein evolution, protein-protein interaction, fibronectin, RECK, affinity, Fc fusion protein

## Abstract

Over the past 3 decades, a diverse collection of small protein domains have been used as scaffolds to generate general purpose protein-binding reagents using a variety of protein display and enrichment technologies. To expand the repertoire of scaffolds and protein surfaces that might serve this purpose, we have explored the utility of (i) a pair of anti-parallel alpha-helices in a small highly disulfide-bonded 4-helix bundle, the CC4 domain from reversion-inducing Cysteine-rich Protein with Kazal Motifs and (ii) a concave beta-sheet surface and two adjacent loops in the human FN3 domain, the scaffold for the widely used monobody platform. Using M13 phage display and next generation sequencing, we observe that, in both systems, libraries of ∼30 million variants contain binding proteins with affinities in the low μM range for baits corresponding to the extracellular domains of multiple mammalian proteins. CC4- and FN3-based binding proteins were fused to the N- and/or C-termini of Fc domains and used for immunostaining of transfected cells. Additionally, FN3-based binding proteins were inserted into VP1 of AAV to direct AAV infection to cells expressing a defined surface receptor. Finally, FN3-based binding proteins were inserted into the Pvc13 tail fiber protein of an extracellular contractile injection system particle to direct protein cargo delivery to cells expressing a defined surface receptor. These experiments support the utility of CC4 helices B and C and of FN3 beta-strands C, D, and F together with adjacent loops CD and FG as surfaces for engineering general purpose protein-binding reagents.

Protein reagents that specifically bind to a target molecule, often another protein, are ubiquitous in biomedical research, clinical diagnostics, and biologic drug therapy (1, 2, 3). The most commonly used binding proteins are antibodies. The high affinity and specificity of antibodies arises from a combination of germline repertoire diversity, error-prone combinatorial gene assembly, and affinity maturation *via* somatic mutation, all driven by Darwinian selection at the level of B-cell survival and proliferation. With the advent of *in vitro* display technologies—such as phage display, ribosome display, and yeast display—to enrich binding proteins from large libraries of sequence variants, antibody-based protein engineering has been expanded by uncoupling the sequence search space from constraints imposed by the immune system, such as suppression of self-reactivity (4).

The same display technologies that have been used for antibody engineering and enrichment have also been applied to a variety of non-antibody protein scaffolds. These are generally small and highly stable proteins, and they include designed ankyrin repeat proteins (DARPins; composed of helices and loops), the 10th fibronectin type 3 domain in human fibronectin (FN3/monobodies/adnectins; a beta-sandwich with three loops at each end), lipocalin (anticalins; a beta-barrel with four loops surrounding a central cavity), and Sso7d (a 7 kDa protein composed of beta strands and one helix) (5, 6, 7, 8, 9). The diverse structures of these scaffolds provide a variety of binding motifs distinct from those used by antibodies.

Binding between antibodies and their targets occurs *via* loops in the complementarity determining regions (CDRs) of the heavy and light chain variable domains, with three CDRs per chain. Camelid-derived single chain antibody variable regions (nanobodies) also use three variable loops for target recognition (10). In contrast, DARPins present a relatively flat binding surface composed of alpha-helical repeats, the anticalin interaction interface consists of widely spaced loops surrounding a central cavity, and the Sso7d-binding surface consists of a three-stranded beta-sheet. As with nanobodies, most work with the FN3 scaffold has introduced variability in three adjacent loops (5, 8). Several studies aimed at expanding the versatility of the FN3 scaffold have found that surfaces beyond the three loops can also be used for target binding (11, 12, 13, 14).

The principal motivation for the present study was to explore the potential utility of two less commonly used structural motifs as general-purpose protein-binding interfaces. In an extension of previous work on the FN3 domain, we chose, as one of these motifs, one of the two FN3 beta-sheet surfaces and introduced mutations in this surface with or without additional mutations in two flanking loops. For the second motif, we chose two adjacent anti-parallel alpha-helices and a short adjoining loop in a small 4-helix bundle domain present in mouse RECK (reversion-inducing Cysteine-rich protein with Kazal Motifs). RECK is a multidomain glycosyl-phosphatidyl-inositol (GPI)-anchored cell-surface protein that was originally identified based on its ability to induce morphological reversion in K-RAS–transformed NIH 3T3 cells (15). At the N terminus of the RECK protein, five small domains, referred to as CC domains, play an essential role in WNT7A and WNT7B signaling in conjunction with a FZD receptor, an LRP5 or LRP6 co-receptor, and GPR124 (16, 17, 18). The 3-dimensional structure of RECK CC domain 4 (CC4) was determined by x-ray crystallography and found to consist of a compact four-helix bundle with three disulfide bonds (19). Sequence comparisons among the five CC domains, as well as comparisons of CC domain sequences across species, show that the CC domain is highly tolerant of sequence variation (19), thus recommending it as a scaffold for engineered diversification.

The second motivation for the present work was to explore useful variations in current methodology related to phage display screening (20), computational and experimental assessment of enriched protein binders, and the application of engineered binding proteins for detecting their targets in the contexts of fluorescent cell staining, viral infection, and protein injection.

## Results

### M13 phage display of RECK(CC4) and FN3

**Figure 1:**
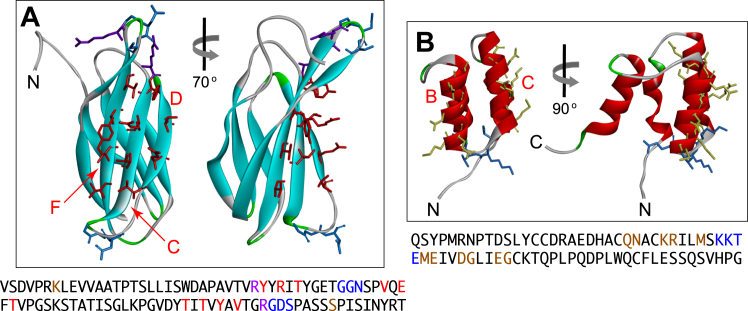
**FN3 and CC4 mutagenesis strategies.***A*, ribbon diagrams of the 10th FN3 domain from human fibronectin (PDB ID 1FNF) showing side chains for the amino acids that were subject to mutagenesis. N, amino terminus. Beta-sheets C, D, and F are labeled with *red* lettering. *Dark red*, amino acid side chains comprising the solvent-exposed surface on one side of the beta-sheet core (beta-sheets C, D, and F). *Blue*, the most proximal amino acid side chains on two loops adjacent to the mutagenized beta-sheet surface (loops CD and FG). The two images differ by a 70-degree rotation about a vertical axis. The amino acid sequence of FN3 is shown below, with mutagenized amino acids color-coded as in the ribbon diagram. Two mutations (D7K and K86S), shown in *brown*, were introduced to stabilize FN3. *B*, ribbon diagrams of RECK CC4 (PDB ID 6WBJ) showing side chains for the amino acids that were subject to mutagenesis. C, carboxy-terminus. The B and C alpha-helices are labeled with *red* lettering. *Yellow*, amino acid side chains comprising the solvent-exposed surface of the B and C alpha-helices. *Blue*, amino acids comprising the loop connecting the B and C helices. The two images differ by a 90-degree rotation about a vertical axis. The amino acid sequence of CC4 is shown below, with mutagenized amino acids color-coded as in the ribbon diagram. RECK, reversion-inducing Cysteine-rich protein with Kazal Motif.

As noted earlier, the 10th FN3 domain from human fibronectin has been used extensively as a scaffold for generating binding proteins, referred to as monobodies (5, 21). To extend existing studies on FN3 surfaces beyond the loops (11, 12, 13, 14) and, more specifically, to explore the utility of randomizing the side chains lining the concave FN3 beta-sheet surface, we created M13 gene 3 phage display libraries in which (1) ten amino acid representing most of the surface of beta-sheet strands C, D, and F were randomized or (2) these ten amino acids were randomized together with three amino acids in each of the nearest two loops (CD and FG), one at each end of the beta-sheet (Fig. 1*A*; (22). To our knowledge, RECK(CC4) (hereafter “CC4”) has not previously been used as a scaffold for protein engineering. As our starting point, we created CC4 libraries in which (1) the eleven amino acids on the adjacent outward-facing surfaces of helices B and C were randomized or (2) these eleven amino acids were randomized and, additionally, the four amino acids in the B-C connecting loop were either mutated from KKTE to GSSG or randomized (Fig. 1*B*).

For both the FN3 and CC4 libraries, we sought to avoid in-frame termination or cysteine codons and, therefore, the codons to be randomized were replaced either with (C,A,G)NN or with (C,A,G)N(C,G). Both strategies eliminate all codons with T in the first position (*i.e.*, the upper quarter of the standard genetic code table), thereby also eliminating codons for phenylalanine, tyrosine, and tryptophan, while retaining codons for the other 16 amino acids (Fig. S1*A*). Next generation sequencing (NGS) of the FN3 and CC4 libraries showed approximately equal representation of the desired nucleotides at each of the randomized positions, as shown for a CC4 library constructed with the (C,A,G)NN codon randomization strategy (Fig. S1*B*). Library complexities was determined to be ∼3 × 10^7^ independent clones based on the percent overlap in read identities between independent pairs of NGS runs (See Experimental procedures).

**Figure 2:**
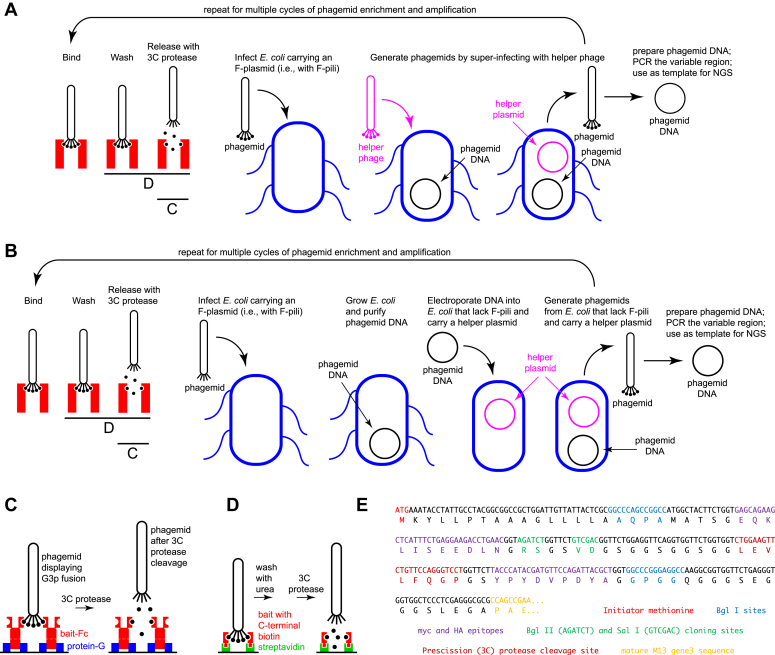
**Phage display work-flow and the M13 gene 3 phagemid expression vector.***A*, conventional protocol for the cycles of phagemid enrichment and amplification. Phagemids are generated by superinfection of F+ *Escherichia coli* with a defective helper phage. The five small *black* balls at the *lower* end of the phage particle represent five copies of the engineered binding domain fused to the N terminus of the gene 3 protein. These bind to the bait (the *red* vertical *rectangle*) and are released following cleavage of an extended linker by 3C protease. The wash and release steps are shown in greater detail in *panels C* and *D*. *B*, modified protocol for the cycles of phagemid enrichment and amplification. Enriched phagemids are recovered by infection of an F^+^*E. coli*, amplified by growing the phagemid (*i.e.*, plasmid) DNA, and then the purified phagemid DNA is electroporated into F^-^*E. coli* that carries a packaging defective helper plasmid. The F^-^*E. coli* carrying both the helper plasmid and the phagemid DNA secretes helper-free phagemid with high yield. Following the final round of amplification, the phagemid DNA is used as a PCR template for NGS. *C*, schematic showing the capture of phagemids with bait-Fc fusion proteins immobilized on protein-G–coated magnetic beads, followed by phagemid release following 3C protease cleavage between the protein-binding domain and the C terminus of the gene 3 protein. *D*, schematic showing urea washing of captured phagemids. The bait proteins carry a C-terminal AviTag and are biotinylated *in vivo*, immobilized on streptavidin-coated magnetic beads, and then used to capture phagemids. After urea washing, phagemids are released by 3C protease cleavage as in (*C*). *E*, sequence at the N terminus of the gene 3–coding region in the modified pADL10-b phagemid vector. The unique Bgl II– and Sal I–cloning sites are flanked on the 5′ side with sequences coding for the *pelB* signal peptide followed by a myc epitope (EQKLISEEDLN) and on the 3′ side with sequences coding for a Prescission (3C) protease site (LEVLFQGP) followed by an HA epitope (YPYDVPDYA).

In an initial series of experiments with CC4 libraries in a conventional M13 gene 3 phage display vector [Fig. 2*E*; a modified version of pADL-10b (Antibody Design Laboratories)] and superinfection with M13 helper phage CM13d3 [a kanamycin-resistant helper phage that is missing gene 3 (Antibody Design Laboratories); the “hyperphage” method of (23)], we observed a several log reduction of phagemid titer with the CC4 phagemid library compared to the starting phagemid vector or the phagemid vector carrying WT CC4 coding sequences. The reduction in titer was also observed when *Escherichia coli* harboring the WT CC4 phagemid were mixed with *E. coli* harboring the CC4 phagemid library. Guessing that reduced phagemid production could be caused by superinfection of the F-plasmid–containing *E. coli* cells by phagemids-encoding mutant CC4 proteins that are deleterious to cell health or viability, we developed an alternative phagemid production workflow in which the phagemid library is introduced by electroporation into DH5alpha cells carrying CM13d3. Since DH5alpha cells lack F-pili, they should be resistant to phagemid infection. With this alternate protocol, we obtained phagemid yields from the CC4 and FN3 libraries of ∼10^10^ to 10^11^ cfu/ml, similar to those observed with the starting phagemid vector or the phagemid vector carrying WT CC4 or FN3 coding sequences. This approach is similar to the “helper cell” strategy developed by Bradbury *et al.* (24, 25), with the additional feature that we have eliminated the possibility of re-infection by using pilus-negative producer cells. For consistency, we also used DH5alpha + CM13d3 as the host for subsequent rounds of phage enrichment.

As diagrammed in Figure 2*A*, in the conventional protocol for M13 phagemid enrichment, an enriched pool of phagemids is recovered by the infection of F-pili–containing *E. coli*, grown in the presence of antibiotic (typically ampicillin) to select for the phagemid genome, and then superinfected at high m.o.i. with a defective helper phage that carries a different antibiotic resistance gene (typically kanamycin). Figure 2*B* shows the alternate protocol used here. As with the conventional protocol, the enriched pool of phagemids is recovered by the infection of F-pili–containing *E. coli* (TG1 in the present instance) and grown in the presence of ampicillin to select for the phagemid genome. The enriched phagemid pool is then recovered as plasmid DNA, which is electroporated into DH5alpha carrying CM13d3 and lacking F-pili. Overnight growth in ampicillin + kanamycin consistently yields high titer phagemids in the medium.

The bait proteins used in the present study were all derived from cell-surface or secreted mouse proteins and were expressed as fusions to human IgG Fc in transiently transfected HEK293T cells. The bait-Fc fusions included only the extracellular domains of the parent proteins (Table S1). In our phagemid enrichment workflow, the bait-Fc fusions were first captured on proteinG magnetic beads, the phagemid library was incubated with the bait-Fc beads, unbound phagemids were washed away with physiologic saline containing 0.1% Tween-20, and bound phagemids were released following proteolysis with 3C protease, which cleaves the CC4-p3 and FN3-p3 fusion proteins just C-terminal to the CC4 or FN3 domain (Fig. 2, *B* and *C*). For higher stringency washing using 4 M urea, the bait proteins were prepared with a C-terminal extension comprising an 8xHis tag, an AviTag, and a rhodopsin 1D4 epitope tag in the place of Fc, and they were secreted as C-terminally biotinylated proteins following co-expression with a endoplasmic reticulum–localized BirA protein (26). The biotinylated baits were captured on streptavidin magnetic beads. We observed that these beads could be washed with 4M or 8M urea at room temperature for tens of minutes without releasing detectable quantities of the biotinylated bait proteins or decreasing the phagemid titer (Fig. 2*D*). In the paragraphs that follow, the sequences named NY1 (against FLRT3) and NY5 (against EPHA4) were derived from the FN3 beta-sheet + two loops library after 4 M urea washes.

### Quantifying phagemid enrichment

**Figure 3:**
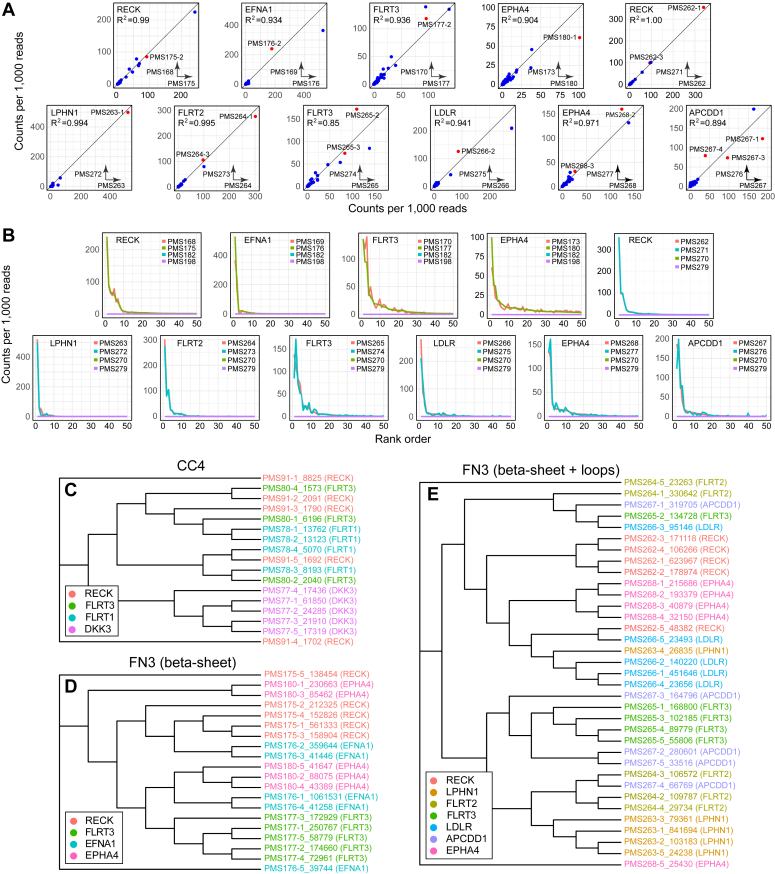
**Enrichment of specific FN3 and CC4 sequences following two rounds of phagemid binding, release, and amplification.***A*, the X and Y axes show NGS read counts (per 1000 reads) for two independent replicates that were processed through two rounds of phagemid capture and release. The names of each experiment are indicated in the inset in the *lower* right of each scatterplot. Each bait consists of all or part of the extracellular domain of the indicated protein fused to human Fc and are listed in the *upper left* of each scatterplot (Table S1). The first four plots (*upper row*, *left four* plots) represent the FN3(beta-sheet) library, and the remaining seven plots represent the FN3(beta-sheet + two loops) library. *Red* symbols represent the phagemid sequences that were subjected to in-depth biochemical characterization. The vast majority of the sequences in the original library are present at less than one read per million reads. *B*, NGS read counts for the 50 most enriched phagemids in each of the 11 pairs of experiments from panel (*A*) are plotted in rank order. Results from four experiments are plotted in each panel. The two experiments listed first (*orange*) and second (*green*) represent the two replicates shown in the corresponding panel in (*A*). The experiments listed third (*blue*) and fourth (*magenta*) represent controls and show the same sets of 50 sequences that are plotted in the *orange* and *green* curves, but with read counts obtained from parallel experiments with a different bait-Fc protein. The two control curves are superimposed and show <1 read count per 1000 reads for each of the 50 sequences. *C*–*E*, dendrograms showing the relatedness at the amino acid level among the most highly enriched phagemid sequences. The horizontal arm lengths within the dendrograms have been quantized to reflect the rank order but not the degree of sequence relatedness. The protein sequences are listed in Table S2. *C*, the CC4 library captured with the four baits listed; (*D*) the FN3(beta sheet) library captured with the four baits listed; and (*E*) the FN3(beta-sheet + two loops) library captured with the seven baits listed.

For each bait, two cycles of phagemid enrichment were performed with duplicate samples and the second round plasmid pools were subjected to NGS. Figure 3*A* shows scatter-plot comparisons of read counts between duplicate experiments for 11 phagemid enrichments: four experiments with an FN3 library in which only the beta-sheet surface was randomized (Fig. 3*A* top row, left four panels) and seven experiments in which the beta-sheet surface and adjacent two loops were randomized (Fig. 3*A*, top row rightmost panel and all panels in the second row). Read counts between duplicate samples are highly correlated, with most data points falling close to the 45 degree diagonal and correlation coefficients >0.85. Although the complexity of each enriched pool of phagemids is high, with ∼10^4^ to ∼10^5^ distinct sequences per sample, nearly all of the sequences are rare (<1 read per 1000 reads) and presumably represent nonspecific contamination. For the same 11 pairs of duplicate experiments, Figure 3*B* shows the distributions of read counts for the top 50 sequences, revealing the small number—typically fewer than five—of substantially enriched sequences. As one measure of the specificity of phagemid capture, each plot in Figure 3*B* also shows, for a different bait, the read counts for the same 50 sequences, and in all cases, these control read counts are at or close to zero.

We expected that a subset of the enriched CC4- or FN3-binding proteins might bind to proteinG, which was covalently linked to the magnetic beads or to Fc, which was part of each bait-Fc protein. Surprisingly, phagemid capture based on binding to proteinG or Fc does not appear to have occurred, as judged by the absence of any CC4 or FN3 sequences that were enriched by more than one bait. This observation suggests that in the context of the bait-Fc+proteinG complex on the bead surface, the Fc domain and proteinG were less accessible for phagemid binding than was the protruding bait domain.

General structural features of binding proteins and their baits—such as the convexity or concavity of surfaces and flexibility of loops—could favor a relatively small number of binding sites on the bait proteins, each with a particular favored geometry for the protein–protein interaction. If this view is correct, then it suggests that, for each bait, the sequences of the enriched CC4- and/or FN3-binding proteins should define a small number of clusters in sequence space, each of which would be predicted to encode proteins that bind the bait with nearly identical locations and geometries. To explore this idea, the relatedness of the most enriched sequences was compared both within and between baits (Fig. 3, *C*–*E*). By visual inspection of the dendrograms of amino acid relatedness, it is apparent that some sequences enriched with a particular bait are not clustered, such as the three CC4 sequences enriched with FLRT3 and the four CC4 sequences enriched with RECK (Fig. 3*C*). However, sequences enriched with some of the other baits cluster in a manner that is far from random, as seen, for example, with the five CC4 sequences enriched with DKK3 (Fig. 3*C*), the four FN3 sequences from the beta-sheet library enriched with FLRT3 (Fig. 3*D*), and the four FN3 sequences from the beta-sheet + two loops library enriched with RECK (Fig. 3*E*), each of which forms a single cluster. The most common pattern is intermediate between these extremes, with a set of sequences enriched by a single bait falling into a small number of clusters. Quantitative analyses of the clustering of the sequences represented in Figure 3, *C*–*E* supports the assessment made by visual inspection (Fig. S2). The sequences of all of the binding proteins analyzed in Figure 3, *C*–*E* and studied in functional assays, as described in the paragraphs that follow, are shown in Tables S2 and S3.

### Tests of the binding proteins

**Figure 4:**
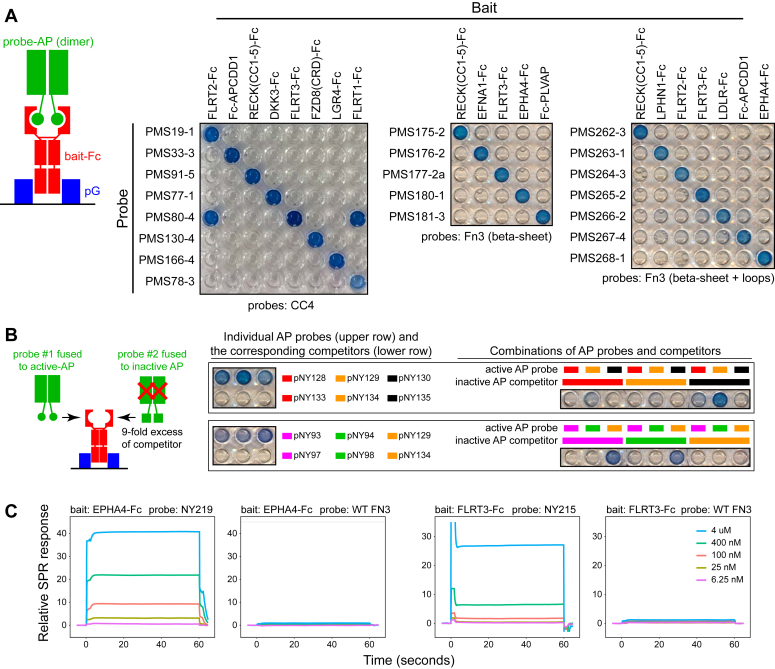
**Biochemical assessment of binding specificity and affinity with purified bait proteins.***A*, *left*, schematic showing bait-Fc fusion proteins immobilized in protein-G–coated wells of a 96-well plate and probed with CC4 or FN3 fusions to hAP. Both the bait and the probe are dimers. *Right*, probe-AP fusion proteins captured by baits immobilized in protein-G–coated 96-well plates. AP activity is visualized with a chromogenic substrate. From *left* to *right*, the probe-AP fusions were derived from CC4, FN3(beta-sheet), and FN3(beta-sheet + two loops) libraries. *B*, *left*, schematic of the competition between eAP fusion proteins and dead-eAP fusion proteins, all of which bind to FLRT3. The schematic is a smaller version of *panel A*. *Right*, Two sets of pairwise competitions among three FN3-eAP fusions and the corresponding FN3-dead eAP fusions. Within each well, the FLRT3-Fc bait was first captured with protein-G and then the indicated fusion proteins were bound and visualized with a chromogenic substrate. The *left* images show two sets of six wells with the individual fusion proteins bound to the FLRT3-Fc bait. For each set of six wells, three FN3 sequences were tested as eAP fusions (*upper row* of three wells) or as dead-eAP fusions (*lower row* of three wells). The three FN3 sequences are color coded (*e.g.*, *red*, *orange*, and *black*). The *right* images show nine wells with the indicated pairwise competition-binding assays. The FN3 sequences used in the eAP probes and dead-eAP competitors are as follows. *Upper* set: *red*, NY1-2; *orange*, NY1-3; *black*, NY1-4. *Lower* set: *magenta*, PMS265-2; *green*, PMS265-3; *orange*, NY1-3. The NY sequences are derived from FN3(beta-sheet + two loops) phagemid enrichment following a 4M urea wash, as described in Experimental procedures and are shown in Table S3. *C*, surface plasmon resonance (SPR) binding assays for two FN3-binding proteins and WT FN3 controls with immobilized EPHA4-Fc (*left*) and FLRT3-Fc (*right*). FN3-MBP fusion protein NY219 carries FN3 sequence NY5-7, and FN3-MBP fusion protein NY215 carries FN3 sequence NY1-3. The concentrations of the FN3-MBP fusions in the mobile phase are color coded as shown in the *right-most box*. eAP, *E. coli* alkaline phosphatase; MBP, maltose-binding protein.

As an initial test of binding protein function, coding sequences for 1 to 3 binding proteins per bait were constructed from synthetic DNA and inserted between DNA encoding a signal peptide and human placental alkaline phosphatase (hAP) for expression in transiently transfected mammalian cells. Serum-free conditioned medium containing the dimeric hAP fusion proteins was used to probe a series of bait-Fc proteins immobilized on proteinG-coated 96-well plates, and the bound hAP fusion proteins were visualized with a colorimetric AP substrate (Fig. 4*A*). For the CC4, FN3 beta-sheet, and FN3 beta-sheet + two loops binding proteins, strong binding signals with the corresponding bait-Fc proteins were observed and, with one exception, binding was not observed with other bait-Fc proteins. Interestingly, the one exception was a CC4-binding protein (PMS80-4) that was enriched with a FLRT3-Fc bait and that also bound to FLRT1 and FLRT2 (Fig. 4*A*). The extracellular domains of the three FLRT family members share between 46% and 60% amino acid sequence identity. Presumably, PMS80-4 binds to a site that is conserved among the three members of the FLRT family.

To develop a simple and semi-quantitative screening approach to assess the relative locations and affinities of binding interactions between a given bait and a series of binding proteins, we have explored competition binding assays with binding protein fusions to active or inactive versions of *E. coli* alkaline phosphatase (eAP). More specifically, we constructed fusions between (i) individual binding proteins and eAP with a C-terminal His-tag (derivatives of pSANG14-3F; (27)) and (ii) individual binding proteins and an otherwise identical eAP-His tag partner in which the eAP active site Serine 102 was mutated to alanine (“dead-eAP”; (28, 29)). Like hAP, eAP is a homodimer (30) and therefore is expected to exhibit enhanced avidity relative to monomer binding. FN3-eAP and FN3-dead-eAP fusion proteins were produced in *E. coli*, purified by Ni-NTA affinity chromatography, and used for competition binding to bait-Fc proteins immobilized on proteinG-coated wells.

In eAP competition experiments, a 9-fold excess of an FN3-dead-eAP fusion was preincubated with the immobilized bait-Fc target prior to incubation with an FN3-eAP fusion (Fig. 4*B*, left schematic). In Figure 4*B*, the left pair of 96-well images shows two sets of six wells with individual AP fusion proteins bound to their FLRT3-Fc bait. For each set of six wells, three FN3 sequences were tested as eAP fusions (upper row of three wells) or as dead-eAP fusions (lower row of three wells). The FN3 sequences are color coded (red, orange, black, etc.). As expected, each of the Fn3-eAP fusion proteins binds the FLRT3-Fc bait and converts the colorless chromogenic substrate to a blue reaction product, whereas the corresponding FN3-dead-eAP fusion proteins fail to produce the blue reaction product. The right pair of 96-well images in Figure 4*B* shows two sets of nine wells with competition binding assays from which several conclusions can be drawn. First, as expected, in competitions between each FN3-binding protein-eAP fusion and an excess of the same FN3-binding protein expressed as a dead-eAP fusion (*e.g.* red with red, orange with orange, black with black), the excess dead-eAP competitor effectively reduces binding by the AP probe. Second, in the upper series, the orange probe (pNY129) generates a weak binding signal in the presence of the red competitor (pNY133) and a strong signal in the presence of the black competitor (pNY135). Consistent with this result, the red and black probes (pNY128 and pNY130) are both largely blocked by the orange competitor (pNY134). Additionally, the red probe (pNY128) generates a weak signal in the presence of the black competitor (pNY135), but the black probe (pNY130) is largely blocked by the red competitor (pNY133). Taken together, these data imply the following rank order of binding strength: orange>red>black. Third, in the lower series, the orange probe (pNY129) generates strong binding signals in the presence of the magenta and green competitors (pNY97 and pNY98), and the magenta and green probes (pNY93 and pNY94) are both blocked by the orange competitor (pNY134). Additionally, the magenta probe (pNY93) is largely blocked by the green competitor (pNY98) and the green probe (pNY94) is largely blocked by the magenta competitor (pNY97). Taken together, these data imply the following rank order of binding strength: orange>magenta∼green. We note that this experiment does not assess whether the different FN3-binding proteins interact with the same, nearby, or distant sites on the target protein, only that the binding interactions exhibit mutual inhibition.

Equilibrium dissociation (K_D_) values for binders and their baits were determined by surface plasmon resonance (SPR) using fusions of individual CC4 or FN3 derivatives to the C terminus of *E. coli* maltose binding protein (MBP) in the mobile phase (Fig. 4*C* and Table 1). The MBP fusion proteins were purified on Ni-NTA resin *via* an N-terminal His-tag. They are presumed to be monomeric since MBP is monomeric and CC4 and FN3 are monomeric. The bait-Fc proteins were immobilized *via* proteinA or proteinG binding. For CC4, K_D_ values were determined for 12 derivatives that bind to APCDD1, including six selected from libraries in which the loop connecting helices B and C was mutagenized; these are designated by the sequence of the four amino acid connecting loop in single letter code (MGME, GGVE, etc). For FN3, K_D_ values were determined for three derivatives that bind to FLRT3 and four derivatives that bind to EPHA4. K_D_ values for this collection of binding proteins are in the low-micromolar range. WT versions of CC4 and FN3 show undetectable binding to the baits.

**Table 1:** SPR affinity measurements of monomeric CC4-MBP and FN3-MBP proteins for their bait-Fc targets

CC4 scaffold			FN3 scaffold		
Binder	Bait	K_D_ (μM)	Binder	Bait	K_D_ (μM)
WT	-	>50	NY200 (WT)	-	ND
PMS33-5	APCDD1	7.78	NY213	FLRT3	2.53
PMS33-1 (MGME)	APCDD1	1.49	NY214	FLRT3	9.45
PMS33-1 (GGVE)	APCDD1	5.05	NY215 (urea)	FLRT3	2.61
PMS33-1 (GGME)	APCDD1	3.17	NY216	EPHA4	2.03
PMS33-2 (GMVR)	APCDD1	3.28	NY217	EPHA4	16.8
PMS92-1 (GMRG)	APCDD1	0.53	NY218 (urea)	EPHA4	4.7
PMS92-1 (GMKG)	APCDD1	0.29	NY219 (urea)	EPHA4	0.42
PMS33-1	APCDD1	0.63			
PMS33-2	APCDD1	1.30			
PMS33-3	APCDD1	3.25			
PMS33-5	APCDD1	0.42			
PMS128-3	APCDD1	8.03			

Binding proteins obtained after enrichment that included a 4M urea incubation are designated with “(urea)”. ND, not determined due to insufficient binding.

### Binding protein fusions to Fc from multiple species and with diverse geometries

**Figure 5:**
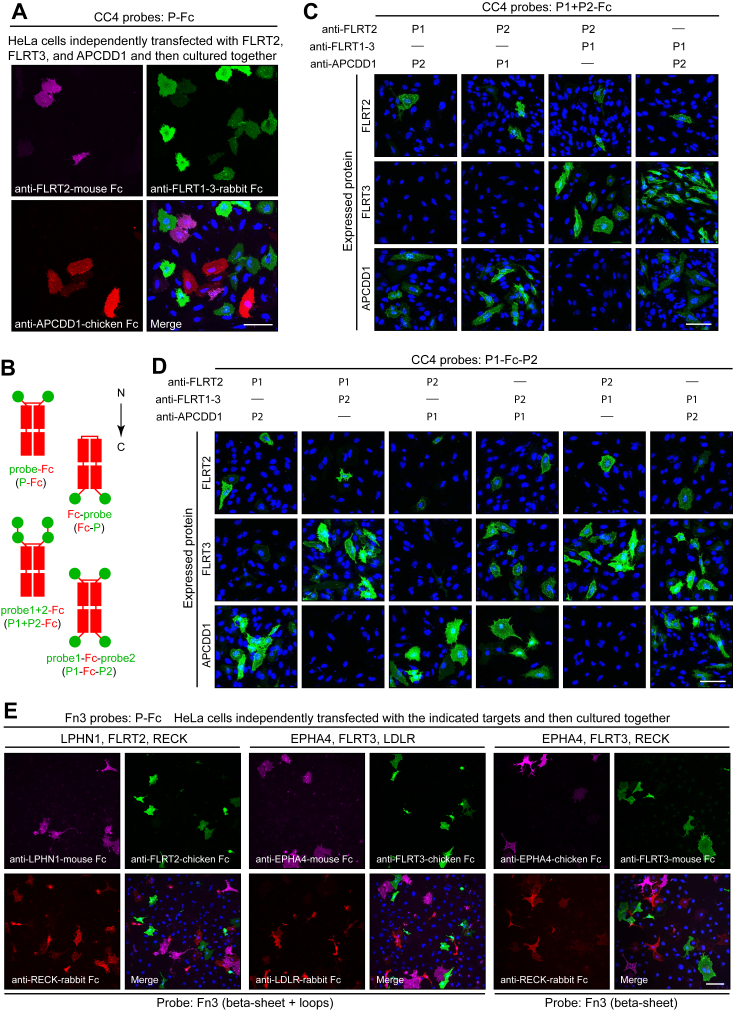
**Immunostaining of full-length cell-surface baits with probes fused to Fc.***A*, HeLa cells independently transfected with full-length FLRT2, FLRT3, or APCDD1 and then cultured together and immunostained with CC4-Fc fusions based on mouse Fc, rabbit Fc, or chicken Fc and visualized with fluorescent anti-mouse, anti-rabbit, or anti-chicken secondary antibodies. The CC4-binding protein that recognizes FLRT1-3 (PMS80-4) binds FLRT3 with higher affinity than FLRT2. *B*, schematic showing the structures of four types of Fc fusion proteins, with protein N- and C-termini oriented as shown by the vertical arrow. *C*, HeLa cells transfected with the indicated full-length bait proteins were immunostained with human Fc fusion proteins consisting of two probes fused to the N terminus of Fc (P1+P2-Fc). For the P1+P2-Fc antibodies in (*C*) and the P1-Fc-P2 antibodies in (*D*), the following CC4-derivatives were used: PMS33-1 for APCDD1, PMS19-2 for FLRT2, and PMS80-4 for FLRT1-3 (with highest affinity for FLRT3). *D*, HeLa cells transfected with the indicated full-length bait proteins were immunostained with human Fc fusion proteins consisting of one probe fused to the N terminus and one probe fused to the C terminus of Fc (P1-Fc-P2). *E*, COS7 cells separately transfected with the indicated full-length bait proteins and then cultured together and immunostained with FN3-Fc fusions based on mouse Fc, rabbit Fc, or chicken Fc, and visualized with fluorescent anti-mouse, anti-rabbit, or anti-chicken secondary antibodies. The following FN3-derivatives were used: PMS268-2 for EPHA4, PMS264-3 for FLRT2, PMS265-2 for FLRT3, PMS263-1 for LPHN1, PMS266-2 for LDLR, and PMS262-1 for RECK. Scale bars in (*A*), (*C*), (*D*), and (*E*) represent 100 μm. RECK, reversion-inducing Cysteine-rich protein with Kazal Motif.

In biomedical research and clinical diagnostics, the most commonly used binding proteins are immunoglobulins. As a result, a large variety of commercially available reagents—such as fluorescent, biotinylated, or enzyme-linked secondary antibodies—are available for detecting immunoglobulins from diverse species. To take advantage of the multiplexing that is possible with these reagents, we constructed mammalian expression vectors that fuse an N-terminal signal peptide and protein-binding domain to the Fc regions derived from mouse IgG, rabbit IgG, or chicken IgY. To test the utility of these Fc fusion proteins, we co-cultured HeLa cells that had been separately transfected with full-length cDNAs coding for FLRT2, FLRT3, and APCDD1 and then immunostained the mixed population with the following CC4-binding protein Fc fusions: anti-FLRT2(PMS19-2)-mouse Fc, anti-FLRT1-3(PMS80-4)-rabbit Fc, and anti-APCDD1(PMS33-1)-chicken Fc, followed by fluorescent secondary antibodies specific for mouse, rabbit, or chicken antibodies (Fig. 5*A*). The resulting pattern of immunostaining corresponds to the specificities of the CC4-derived–binding proteins determined by AP binding (Fig. 4*A*). Similar results were obtained with COS cell transfected with EPHA4, FLRT2, FLRT3, LPHN1, LDLR, and RECK and immunostained with FN3-binding protein Fc fusions with mouse, rabbit, and chicken Fc against these baits (Fig. 5*E*).

Recombinant Fc fusions are not constrained by the natural geometry of immunoglobulins, with a single antigen-binding domain at the N terminus of each polypeptide chain, and thus various N- and C-terminal fusion geometries have been explored as a strategy to increase the number of binding domains per polypeptide (*e.g.*, (31)). To enhance avidity or to generate dual specificity-binding reagents, we have (i) fused two CC4-derived binding domains to the N terminus of human Fc (probe1+2-Fc) and (ii) fused one CC4-derived binding domains to the N terminus and one to the C terminus of human Fc (probe1-Fc-probe2) (Fig. 5*B*). In both double binding protein configurations, immunostaining of transiently transfected cells showed specific binding of the anti-FLRT2, anti-FLRT1-3, and anti-APCDD1 CC4 binding proteins (Fig. 5, *C* and *D*), consistent with the specificities determined by AP binding (Fig. 4*A*).

### Adeno-associated viruses with VP1-FN3 fusions that confer target specificity

Over the past 2 decades, adeno-associated virus (AAV) has emerged as a leading viral vector system for human gene therapy and as an indispensable tool for neuroscience research (32, 33, 34). A major technical challenge in the AAV field has been the identification of capsid variants that confer cell-type–specific infectivity. Several groups have used random mutagenesis of small unstructured peptide insertions in a protruding loop in the VP1 capsid protein together with cycles of infection and genome amplification to identify sequences that favor infection of specific cell types (33, 35, 36, 37, 38). An alternative, and more targeted, strategy is to insert a binding protein of known specificity into one of the capsid loops, as described for nanobody insertion into the GH2/GH3 loop of the VP1 capsid protein (39). The resulting AAV preferentially infects cells displaying the cell surface protein targeted by the nanobody.

**Figure 6:**
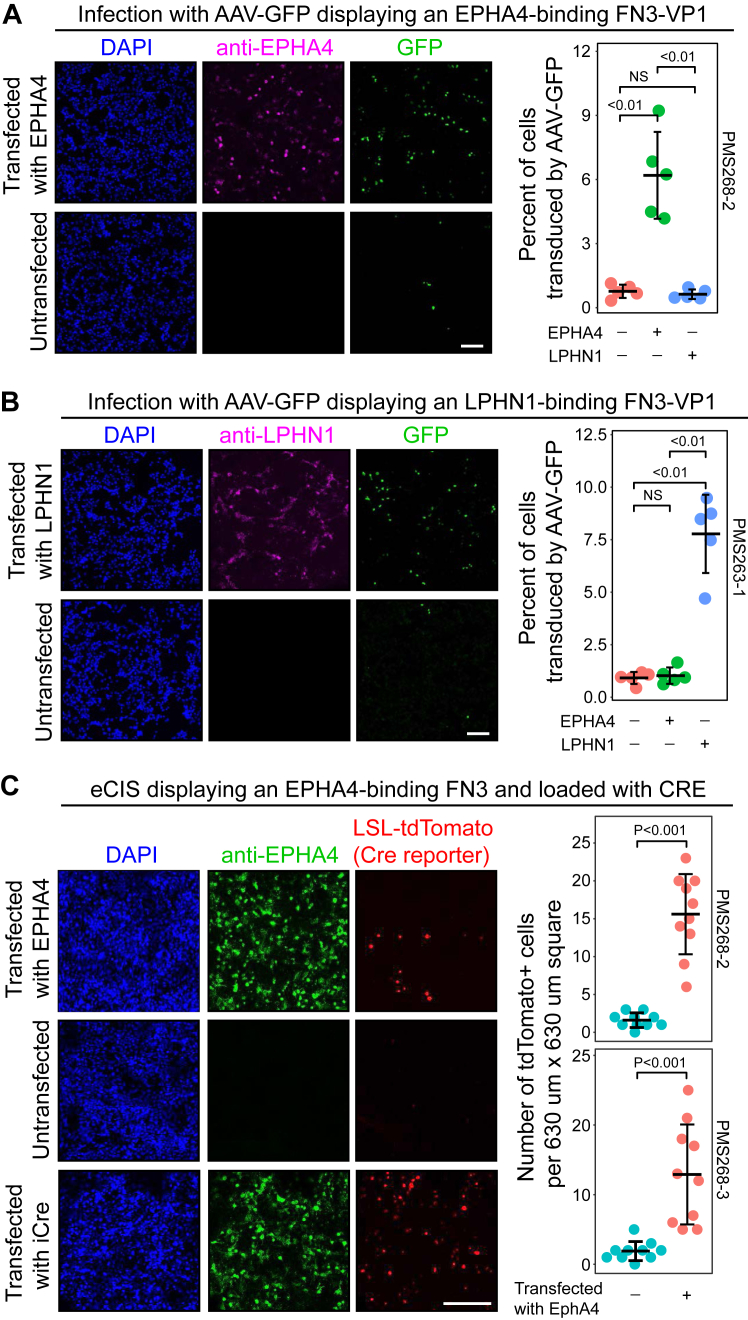
**Engineering FN3-binding protein specificity for viral infection and protein injection.***A* and *B*, HEK293T cells infected with GFP-expressing AAV viruses displaying FN3 insertions in VP1 with specificity for the extracellular domain of EPHA4 (*A*) or LPHN1 (*B*), corresponding to FN3 sequences PMS268-2 and PMS263-1, respectively. Plots show the percent of cells transduced by the virus from five microscope fields with an average of ∼900 cells per field. *C*, injection of CRE recombinase into HEK293T cells by bacterial extracellular contractile injection system (eCIS) particles displaying either of two EPHA4-binding FN3 domains inserted into the tail fiber protein Pvc13. *Left*, HEK293T cells were transfected with a loxP-stop-loxP-tdTomato reporter plasmid (LSL-tdTomato) with or without transfection of an EPHA4 expression plasmid and then incubated 1 day later with eCIS particles displaying the EPHA4-binding FN3 PMS268-2 (*upper two rows*). As a positive control, HEK293T cells were transfected with the LSL-tdTomato reporter plasmid, an EPHA4 expression plasmid, and a CRE-recombinase expression plasmid (iCre) (*lower row*). *Right*, quantification of tdTomato-expressing cells, with or without EPHA4 expression, following incubation with eCIS displaying either of the two EPHA4-binding FN3 domains (PMS268-2 or PMS268-3). Statistical values are presented as mean ± SD. The Wilcoxon rank sum test was used to measure statistical significance. Scale bars in (*A*–*C*) represents 100 μm. AAV, adeno-associated virus.

To extend the domain insertion approach to the FN3 scaffold, we generated GFP-expressing AAVs with anti-LPHN1 and anti-EPHA4 FN3 variants inserted into the GH2/GH3 loop of the VP1 capsid protein and then used them to infect either untransfected HEK293T cells or HEK293T cells transfected with expression vectors coding for full-length LPHN1 or EPHA4. At a multiplicity of infection that transduced ∼10% of the transfected cells, each of the AAV preparations displayed a 5-10-fold preference for cells expressing the receptor to which the displayed FN3 protein binds (Fig. 6, *A* and *B*). In the future, it will be interesting to explore derivatives of such vectors in which VP1 surface residues that mediate uptake *via* ubiquitously expressed receptors are mutated. For example, R585 and R588, located in one of the VP1 loops, appear to mediate binding and infection *via* heparin sulfate proteoglycans, and this interaction can be reduced by mutating these two arginines to alanines. Reducing infection *via* ubiquitous receptors should increase the ratio of specific to nonspecific infection.

### An eCIS with FN3 fusions that confer target specificity

Numerous bacteria produce one or more extracellular contractile injection systems (eCIS) to introduce bacterially produced proteins into target cells. The eCIS consists of a syringe-like structure that resembles a bacteriophage tail. Proteins to be injected are loaded into the eCIS particle during its assembly in the bacterial cell, the eCIS particle is secreted into the medium, and, upon binding to the surface of a target cell, the contents of the eCIS particle are injected (40). Highly specific targeting of eCIS particles to mammalian cells expressing a specific cell-surface protein was recently described by (41). Used the *Photorhabdus* eCIS system, Kreitz *et al* fused various epitope tags or binding domains to the eCIS tail fiber protein (Pvc13) and observed that the resulting eCIS particles were then programmed to deliver their cargo to cells expressing a cell-surface binding partner for the inserted peptide or protein.

To extend the observations of (41), we asked whether FN3 domains engineered to bind cell surface proteins could be fused to the *Photorhabdus* Pvc13 protein and assembled into eCIS particles to deliver a CRE-recombinase payload into HEK293T cells expressing the cognate cell-surface protein. FN3 domains recognizing RECK, LPHN1, or EPHA4 were tested, and CRE delivery was monitored with a loxP-stop-loxP (LSL)-tdTomato reporter. In these experiments, we observed no significant increase in CRE-mediated recombination with RECK and LPHN1 targets. By contrast, two different FN3 sequences that bind to EPHA4 conferred a ∼10-fold increase in the number of cells showing LSL-tdTomato reporter activation compared to control cells not expressing EPHA4 (Fig. 6*C*). This frequency of tdTomato-expressing cells is ∼20% the level observed upon transfection with a CRE-expressing plasmid (Fig. 6*C*). Given that CRE acts as a tetramer to effect recombination between two loxP sites (42), those cells with reporter activation following incubation with eCIS particles likely received many copies of CRE.

## Discussion

The experiments presented here demonstrate that diversified derivatives of the solvent-exposed surface of the B and C helices (with or without the connecting loop) of the RECK CC4 domain and the solvent-exposed surface of the C/D/F beta-sheet (with or without the two adjacent loops) of FN3 have utility as general-purpose protein-binding interfaces. This study also demonstrates the use of (i) NGS to rank order and cluster phagemid-encoded protein binders following enrichment by bait capture, (ii) protein binding with active AP fusions and competition with inactive AP fusions to assess specificity and relative affinities, (iii) immunostaining with a variety of binding protein-Fc fusions, including fusions to Fc from different species and Fc fusions with one or two binding domains and with different Fc fusion architectures, (iv) FN3-binding protein insertion into VP1 of AAV to direct AAV infection to cells expressing a defined surface receptor, and (v) FN3 binding protein insertion into the Pvc13 tail fiber protein of an eCIS particle to direct protein cargo delivery to cells expressing a defined surface receptor.

The engineered protein-binding surfaces explored here are distinct from those that are most commonly used. Unlike the binding surfaces of antibodies, nanobodies, monobodies, DARPins, and anticalins, which are all based on three or more variable loops, the surfaces explored here were centered around two largely planar structures: two anti-parallel helices (RECK CC4) and a beta-sheet (FN3). At present, it is not known whether the set of binding sites on the bait proteins used here differ in any systematic way from the binding sites on these proteins that are recognized by other natural or engineered binding proteins. If that were the case, it might recommend the use of scaffolds based on anti-parallel helices or beta-sheets as one approach to diversifying the universe of interactions accessible to engineered binding proteins.

In the paragraphs that follow, we consider ways in which the reagents and methodologies used here could be further modified and improved.

Since its introduction in the 1990s, the FN3 scaffold has been modified with the goal of enhancing its stability so that it can accept an ever greater number and diversity of sequence variants (43, 44, 45). Using one of the ultra-stable FN3 derivatives as a starting scaffold might permit isolation of a wider range of sequence variants than was observed in the present study. Another variable that could be explored in conjunction with increasing the stability of the FN3 scaffold is the use of different signal peptides in the phage display vector. In a quest for more efficient phagemid display of the highly stable DARPin scaffold (46), it was discovered that translocation of rapidly folding and highly stable proteins, such as DARPins, across the *E. coli* inner membrane occurs more efficiently with signal peptides that engage the cotranslational signal recognition particle (SRP) pathway rather than the posttranslational Sec pathway. More recently (47), it was showed that a derivative of the *pelB* signal peptide could outperform by more than ten-fold both the WT *pelB* signal peptide, which uses the Sec pathway, and the *DsbA* signal peptide, which uses the SRP pathway, when tested with a DARPin cargo. These data suggest that exploring alternative signal peptides for phage display may increase the efficiency of this part of the pipeline.

The CC4 scaffold is distinctive in two respects: it is a 4-helix bundle and it has a high density of disulfide bonds (three disulfides within a total length of 65 amino acids; (19)). Other scaffolds comprised primarily of alpha-helices include Affibodies (a 3-helix bundle) and Cytochrome b562 (a 4-helix bundle) (48). A third alpha-helical scaffold, based on a 4-helix bundle protein from *Bacullus halodurans*, has been engineered to bind Frizzled receptors (49). Highly disulfide bonded scaffolds that have been used for protein engineering include the “knottins”, a diverse family of secreted mini-protein with lengths of 25 to 35 amino acids that function as toxins and protease inhibitors (50), and the Kunitz domain family, protease inhibitors of ∼60 amino acids in length with three disulfide bonds (51). Our experience thus far with the CC4 domain suggests that it displays well on phagemids, but when expressed as a secreted AP fusion protein in HEK293T cells, ∼40% of the B and C helix variants had yields that were lower than the yield of WT CC4. Moreover, when expressed as an MBP fusion protein in *E. coli* SHuffle cells (a mutant strain with a cytosolic redox potential that is permissive for disulfide bond formation (52);), >80% of the B and C helix variants showed markedly lower yields than the WT CC4 parent. In the future, it may be of interest to explore other RECK CC domains as parent sequences for protein engineering. The five RECK CC domains (CC1-CC5) have the same pattern of six cysteines and they share 17 to 29% amino acid identity and 30 to 49% amino acid similarity, implying that they adopt nearly identical tertiary structures but with broad tolerance for sequence substitutions (19). RECK proteins from other species could also provide additional starting sequences.

Another variable that has received substantial attention in the context of antibody CDR and FN3 mutagenesis is the ratio and identity of amino acids within the randomized regions. In the most extreme examples, restricting the introduced amino acids within the CDR or FN3 loops to Y, S, and G or to Y and S was compatible with the identification of high affinity binders (53, 54, 55, 56, 57). Less stringent mutagenesis strategies that feature a high abundance of Y, S, and G and lower abundances of other amino acids have also been used (5). The CC4 and FN3 libraries described here were constructed with a very different mutagenesis strategy: for each randomized region, a single degenerate oligonucleotide was synthesized with a mixture of codons that eliminates F, Y, C, W, and the three stop codons. Thus, the strategy employed here eliminates the amino acid (Y) that appears to be the most useful in the context of CDR randomization, as well as all other aromatic residues except for histidine. It will be interesting to explore the utility of the Y/S/G strategy or less extreme versions of that strategy, in the context of alpha helical surface randomization and beta-sheet randomization, as studied here.

Looking forward, analyses of the interactions between binding proteins and their targets will likely benefit from computational predictions of protein–protein interfaces, especially for those targets for which accurate 3-D structures are available. At present, the gold standard for defining protein–protein contacts is a high resolution X-ray crystal structure of the complex, an approach that cannot easily be scaled to high throughput. Recent advances in the prediction of protein structure and protein–protein interactions suggest that high accuracy and high-throughput computational predictions of engineered proteins and their binding interactions will soon be available (58, 59, 60, 61). These methods should substantially accelerate the identification and characterization of engineered binding proteins.

## Experimental procedures

### Phagemid library construction

The phagemid vector is a derivative of pADL-10b, an ampicillin resistant plasmid that expresses a *pelB* signal peptide-gene 3 fusion protein and has unique cloning sites for the insertion of an ORF immediately after the *pelB* signal peptide (Antibody Design Laboratory). We have not explored the utility of alternate signal peptides, in particular those that direct the displayed protein-gene 3 fusion protein to the cotranslational SRP pathway rather than the posttranslational Sec pathway, as described by (46). pADL-10b was initially modified as shown in Figure 2*E*, with unique Bgl II and Sal I cloning sites. Other derivatives have unique Pst I and Sal I cloning sites. For library construction, the starting vector, with unique Pst I and Sal I cloning sites, was modified by first inserting a Pst I–Sal I DNA segment with a unique internal EcoR I site. The library insert was prepared by PCR amplifying four overlapping synthetic DNA segments (two sense and two antisense), ranging in length from 75 bases to 120 bases, that, when assembled by PCR, code for RECK CC4 or FN3. The synthetic DNA segments had either two, three, or four different bases incorporated at predetermined locations, as shown in Fig. S1, and the final PCR product had Pst I and Sal I sites at its 5′ and 3′ ends, respectively.

The following two-step ligation procedure was devised for library construction, described here for cloning between unique Pst I and Sal I sites. (i) The vector and the PCR-amplified insert were cut with Pst I, gel purified, and ligated at high concentration and at a mole ratio of 10 to 20 inserts per vector. The products of that ligation should consist largely of insert dimers and vectors with inserts ligated to each end. (ii) The ligation products were cut with Sal I, run on a preparative agarose gel, and the DNA segments at the size of vector + insert were purified and ligated at low concentration (<20 μg/ml) to favor cyclization. (iii) The ligation products were cleaved with EcoR I (located between Pst I and Sal I sites in the starting plasmid) to linearize any DNA molecules in which the vector segment that had not been cleaved with both Pst I and Sal I. (iv) The cyclized library was electroporated into electro-competent TG1 cells (Intact Genomics) with a Bio-Rad GenePulser Xcell. Libraries of ∼3 × 10^7^ independent clones were obtained from ∼5 μg of vector and ∼5 μg of insert.

To estimate library size, the percent overlap in read identities was determined between independent pairs of NGS runs obtained from the starting libraries. For each library, the calculation was based on 2 to 4 sequencing runs with 3 to 10 million reads for each run. At this sampling depth, most members of the library are represented by either 0 or 1 reads. As an example for one CC4 phagemid library, two independent sequencing runs gave 9,728,555 and 8,320,509 sequences with 2,950,633 sequences in common, leading to an estimate of 27,433,615 independent clones (with a 95% confidence interval of 27,412,640–27,454,623).

### Bait construction and production

Baits consisted of part or all of the extracellular domains of the mouse versions of cell surface or secreted proteins fused to human IgG1 Fc, either N-terminal to the hinge region or at the C terminus (62). For baits fused at the N terminus of Fc, the signal peptide was derived from the bait protein, and the following linker separated the bait from Fc sequences: GAPGPRTDLTTAAPSPPRRLPPPPPPKLGGG (derived from restriction sites and sequences coding for the mouse FZD8 linker immediately C-terminal to the cysteine-rich domain). For the following proteins, the bait-Fc included the entire N-terminal extracellular domain: EFNA1, EPHA4, FLRT1, FLRT2, FLRT3, LDLR, LGR4. For RECK(CC1-5), LPHN1, and FZD8(CRD), the bait-Fc included only part of the extracellular domain. For DKK3, a secreted protein, the bait-Fc included the entire mature protein. For APCDD1 and PLVAP, the bait included the entire extracellular domain and was fused to the C terminus of Fc. The regions of each bait protein used are shown in Table S1. The two fusions with the bait C-terminal to Fc uses the mouse FZD8 signal peptide for secretion. For EPHA4, FLRT3, RECK(CC1-5), and LPHN1 baits, an analogous set of secreted proteins with an AviTag (GLNDIFEAQKIEWHE), an 8xHis tag, and a rhodopsin C-terminal 1D4 epitope tag (SKTETSQVAPA) fused to the C-terminus in place of Fc were also constructed.

Bait-Fc or Fc-bait proteins (hereafter, simply “bait”) were produced as secreted proteins in serum-free medium (SFM) by transient transfection of HEK293T cells. Two days after transfection, serum-free conditioned medium (SFCM) was harvested, aliquoted, and stored at −80 °C. Protein yield and purity were estimated by capturing 300 to 500 μl of SFCM on proteinG magnetic beads, releasing the captured protein in SDS sample buffer at 95 °C, resolving the bait-Fc protein by SDS-PAGE, and comparing the Coomassie Blue staining intensity with a dilution series of bovine serum albumin (BSA). Yields were typically 1 to 5 μg/ml of SFCM, and purity after proteinG capture was typically >90%.

For production of C-terminally biotinylated proteins, a plasmid encoding the bait-AviTag-8xHis-1D4 fusion and pDisplay-BirA-ER, a mammalian expression vector encoding an ER-localized derivative of BirA (Addgene #20856; (26)), were co-transfected into HEK293T cells. One day later, the medium was replaced with SFM containing 100 μM biotin. Two days later, the SFCM was harvested and 10 mls SFCM was incubated with 250 μl pre-washed Ni NTA resin for 2 h at room temperature. The resin was washed twice to remove free biotin and then incubated with 2.5 mls of 500 mM imidazole with gentle rotating for 2 h at room temperature. The resin was pelleted at 3000*g* for 5 min and the supernatant with eluted proteins was stored in aliquots at −80 °C. Protein yields were estimated by capturing 50 μl of the eluted material on Streptavidin magnetic beads, releasing the captured protein in SDS sample buffer at 95 °C, resolving the bait-Fc protein by SDS-PAGE, and comparing the Coomassie Blue staining intensity with a dilution series of BSA.

### Phagemid enrichment

In a typical phagemid capture experiment, each capture was performed in duplicate and five baits were used, that is, 2 × 5 = 10 samples were processed in parallel. Together with duplicate samples of the starting phagemid library, this generates 12 samples for NGS, which can be accommodated in one lane using the 12 Illumina bar codes listed below under “Next Generation Sequencing”. For each bait, two rounds of phagemid enrichment were performed. As a positive control for testing and optimizing the phagemids enrichment protocol, ProteinG magnetic beads were coated either with anti-myc mAb or with an irrelevant mAb (anti-rhodopsin B6-30) and used to capture WT RECK CC4 phagemids, which display the myc epitope at the N-terminus of the gene 3 protein (Fig. 2*E*). A single round of enrichment yielded ∼1000-fold more phagemid particles with the anti-myc mAb than with the irrelevant mAb.

Although some protocols recommend concentrating phagemid libraries by PEG precipitation prior to the first round of capture, this approach risks generating phagemid aggregates that could bind non-specifically and, therefore, we have avoided it. In a typical first-round phagemid capture reaction, 10^10^ to 10^11^ phagemids/ml in 1.5 ml of bacterial growth medium are mixed with bait-coated magnetic beads. With a library complexity of ∼3 × 10^7^, each member of the library was represented, on average, by 500 to 5000 phagemid particles.

The following solutions were prepared and stored frozen: (i) Tris buffered saline (TBS; 10 mM Tris, pH = 7.0; 150 mM NaCl); (ii) 10X TBS with 1% Tween-20 (10X TBST); (iii) 5% BSA; and (iv) TBS with 0.1% Tween-20 and 0.1% BSA (TBSTB). Twenty five microliters of ProteinG magnetic beads (30 mg/ml, as supplied by Thermo Fisher Scientific) was used per pair of duplicate phagemid capture reactions. ProteinG magnetic beads (125 μl; sufficient for 10 captures) were washed 5 times with PBS, and then 20% of the beads were added to 0.5 mls of the appropriate bait in SFCM in a 1.5 ml tube, and the tube was rotated end-over-end overnight at 4 °C. The next day, the SFCM was removed and each aliquot of bait-Dynabeads was washed three times with PBS, once with TBSTB, and resuspended in 100 μl TBSTB.

In a 2 ml screw-capped tube, 170 μl of 10X TBST and 30 μl 5% BSA were added to 1.5 ml of high titer phagemid stock in bacterial growth media (typically 10^10^–10^11^ phagemids per ml), which brings the phagemid stock solution to 1X TBSTB. Fifty microliters of the resuspended magnetic beads with bound bait in TBST was added to each tube and the tubes were rotated end-over-end overnight at 4 °C. The next day, the phagemid solution was removed and the magnetic beads with bound bait were resuspended in 400 μl TBSTB and transferred to a new 1.5 ml tube to minimize contamination from residual phagemids on the top or walls of the original tube. The magnetic beads with bound bait and phagemids were washed three times with 0.5 mls TBSTB, and then resuspended in 5 μl TBSTB.

His-tagged PreScission (3C) protease was prepared as described (63) and stored in aliquots at −80 °C at a concentration of ∼1 mg/ml. 3C protease was diluted 1:1 in TBS and then 6 μl of the diluted protease was added to each tube containing magnetic beads in 5 μl TBSTB. The tubes were maintained at room temperature and, to intermittently resuspend the magnetic beads, gently vortexed at intervals of 15 to 20 min over 3 h. At the end of the 3 h incubation, the magnetic beads were removed with a magnet and the bead-free solution containing released phagemids was used to infect 1 ml of exponentially growing TG1 cells at 37 °C. After a 45 min incubation with vigorous circular rotation to promote phagemid absorption and expression of the *AmpR* gene, the 1 ml culture was diluted into 20 mls Superbroth containing 50 mg/ml ampicillin. A 20 μl sample was removed and plated on ampicillin plates to assess the number of transductants, and the remaining culture was grown overnight at 37 °C. After overnight growth, two samples of 1 ml each were saved from each culture as 10% DMSO freezes at −80 °C, and the remaining 18 mls was used to prepare plasmid DNA.

To prepare phagemids, purified plasmid DNA from each overnight TG1 culture was electroporated into DH5alpha cells carrying CM13d3, an M13 helper plasmid that confers kanamycin resistance and is missing gene 3 (Antibody Design Laboratories). After the electroporated bacteria were grown overnight in 70 μg/ml kanamycin and 50 μg/ml ampicillin at 32 °C in a non-baffled flask, the cells were spun down and samples saved as 10% DMSO freezes at −80 °C, and the phagemids in the supernatant were titered by infecting TG1 cells, as described above. In our hands, phagemid yields are better with overnight growth at 32 °C compared to 37 °C. Typically, 10^10^ to 10^11^ phagemids per ml were produced, as determined by counting ampicillin resistant colonies. The number of kanamycin resistant colonies, derived from transduction of CM13d3 phage particles, was >1000-fold lower.

After the second round of enrichment, miniprep DNA was prepared from the overnight TG1 culture for use as a PCR template for NGS.

### Urea washing for increasing the stringency of phagemid enrichment

To identify FN3 derivatives with higher affinity binding to the bait proteins, we added a 15-min incubation with 4 M urea. This step likely has no effect on phage viability because, in preliminary experiments, we observed no effect on phage viability of a 15-min room temperature incubation in either 4 M or 8 M urea. After the phagemids were captured by bait-coated magnetic beads, and the beads washed with TBSTB, the bead+bait-bound phagemids were incubated with 4 M urea for 15 min at room temperature with gentle rotation, washed 3 × 1 ml in TBSTB, and then incubated with 3C protease.

### Next generation sequencing

For PCR amplifying the 12 samples, a single Illumina sense primer was paired with a set of 12 Illumina antisense primers with 6-base bar codes. The sequences of the sense primer and one of the 12 antisense primers used for amplifying the randomized region of the CC4 libraries follow. Sense Primer (with the UMI represented by Ns): AATGATACGGCGACCACCGAGATCTACACTCTTTCCCTACACGACGCTCTTCCGATCTNNNNNNNNNNACTGATAGTTTATATTGCTGT.

One of the 12 antisense primers (with the 6-base bar code represented in italics): CAAGCAGAAGACGGCATACGAGAT*CGTGAT*GTGACTGGAGTTCAGACGTGTGCTCTTCCGATCTAATGGAAATTGGCTTGCTGCT.

Sequencing templates were prepared by amplifying 250 ng of starting plasmid with 12 cycles of PCR using Phusion High Fidelity DNA Polymerase (New England BioLabs). PCR products were purified (Qiaquick) and sequenced together on one lane of an Illumina NovaSeq.

Due to the uniformity of the sequences flanking the randomized codons, the following modifications were made to the standard Illumina flow-cell protocol: (i) libraries were seeded into the flow cell at several-fold lower than standard density and (2) PhiX174 genomic DNA was spiked into the library at a level of 50%.

### Sequence analysis

The cut adapt program (https://doi.org/10.14806/ej.17.1.200, version 4.3) was used to trim the reads, removing sequences that matched WT FN3 or CC4 (64, 65). Only trimmed reads were kept. For FN3 reads, where paired end sequencing was used to sequence the two regions that were randomized, the seqkit (version 2.4.0) program was used to obtain the reverse complement of read 2. The two reads were joined *via* their read names, using the unix ‘join’ command, and the intervening FN3 sequences were added to complete the sequence. The number of occurrences of each sequence was counted using an awk script and the sequences were ranked in descending order by the number of occurrences. Further analyses and plotting were performed with R (version 4.3.0) using Studio IDE (66). The sequence logo image (Fig. S1*B*) was produced with the ggplot2 (version 3.4.2) extension ggseqlogo (version 0.1) using the most abundant one million sequences. Scatter plots and line plots were made with ggplot2.

Dendrograms (Fig. 3, *C*–*E*) and scatter plots of percent identity (Fig. S2) were produced using ClustalW in MacVector, with the following parameters: (i) pairwise alignment mode: slow, (ii) pairwise alignment parameters: open gap penalty = 10.0 and extend gap penalty = 0.1, and (iii) similarity matrix: gonnet. For Fig. S2, quantitative analysis of sequence clustering in the dendrograms in Figure 3, *C*–*E*, the percent identity was determined for each pairwise amino acid sequence comparison (excepting each sequence with itself), and the statistical significance was calculated for comparisons among the sets of sequences enriched by different baits.

### AP, Fc, and MBP fusion vectors

For expression of secreted human AP-fusion proteins from HEK293T cells, the coding region for hAP minus the signal peptide and GPI anchor was fused C-terminal to the FZD8 signal peptide. The mutated CC4 or FN3 coding regions were inserted between the signal peptide and hAP. The CC4 and FN3 inserts were assembled by PCR with synthetic DNA based on the sequences of enriched phagemids.

Vectors for the expression of Fc fusions in HEK293T cells were cloned in pRK5 and used the mouse FZD8 signal peptide followed by genomic regions (*i.e.* exons+introns) coding for one of the following: (i) human IgG1 Fc with a 20 amino acid spacer between the insert and the start of the hinge region, (ii) mouse IgG-2a Fc, (iii) chicken IgY Fc, and (iv) rabbit Fc. The human IgG1 sequences were a gift of Dr Brian Seed (62). Mouse, chicken, and rabbit Ig sequences were PCR amplified from genomic DNA. Derivatives of the human Fc fusion vector were created with (i) two sets of cloning sites between the signal peptide and the hinge for the P1-P2-Fc arrangement or (ii) with cloning sites immediately 5′ of the Fc stop codon for the Fc-P arrangement. P1-Fc-P2 expression plasmids were constructed by joining together P-Fc and Fc-P segments *via* a unique Xma I site within the human Fc gene.

FN3 fusions to the N-terminus of *E. coli* alkaline phosphatase (eAP) were generated in pSANG14-3F (Addgene #39265; (27)). The inactive eAP derivative (“dead eAP”) vector was generated by mutating the active site Ser102 of *E. coli* AP to Ala (28, 29). Synthetic DNA encoding FN3 sequences identified in phagemid screens were cloned into the pSANG-eAP and/or pSANG-dead-eAP vectors.

For the SPR assay, FN3 and CC4 sequences were cloned into and expressed from a pET 11d vector downstream of a 8xHis-tagged MBP ORF. All standard PCR amplifications were performed with Phusion DNA polymerase (New England Biolabs), and the final plasmid inserts were sequenced to rule out spurious mutations.

### CC4 and FN3 AP and MBP protein production

For production of AP fusion proteins in mammalian cells, secreted proteins were produced in SFM by transient transfection of HEK293T cells, as described under “Bait construction and production” for bait-Fc proteins. AP activity was tested by incubation of 1 to 5 μl of SFCM or purified *E. coli* AP fusion proteins with the BluePhos AP chromogenic substrate system (Kirkegaard and Perry Laboratories).

*E coli* expression of CC4 constructs was in SHuffle cells (T7 Express with lysY; New England BioLabs #C3030J). *E coli* expression of FN3 constructs was in BL21. *E. coli* carrying MBP, eAP, or dead-eAP expression plasmids was cultured in superbroth media with ampicillin at 37 °C. When the A_600_ reached 0.4, IPTG was added to a final concentration of 100 μM to induce protein expression overnight with vigorous aeration at room temperature. The next day, cells were pelleted and lysed with Bugbuster cell lysis buffer (Sigma-Aldrich). After removing cell debris by centrifugation at 13,000*g* for 10 min at 4 °C, the supernatant was collected and diluted 1:1 with binding buffer and incubated with pre-washed Ni-NTA resin for 2 h at 4 °C. The resin was washed twice and then rotated with elution buffer containing 500 mM imidazole for 2 h at 4 °C. The resin was pelleted at 3000*g* for 5 min and the supernatant with eluted proteins were stored in aliquots at −80 °C. Purity was estimated by SDS-PAGE.

### 96-Well AP binding assay

One hundred microliters of SFCM containing the appropriate bait-Fc fusion proteins was added to each well of a ProteinG-coated 96 well plate (Thermo Fisher Scientific) and incubated overnight at 4 °C. The following day, each well was incubated for 3 h at room temperature with 300 μl PBS with 1% BSA, washed three times with SFM containing 0.1% BSA, and incubated overnight at 4 °C with 100 μl of SFM containing 0.1% BSA and a 1:10 dilution of the AP probe in SFCM. The next day, the wells were washed five times with 300 μl ice-cold PBS with 1% TritonX-100, and then 50 ul of the BluePhos AP chromogenic substrate was added per well and the tray incubated with gentle horizontal rotation at room temperature until the blue reaction product was observed.

### Dead AP competition experiments

After the bait-Fc protein (in SFCM) was bound to the proteinG-coated wells of a 96-well plate, the wells were blocked with 1% BSA overnight at 4 °C and then washed three times with SFCM containing 0.1% BSA. Forty-five microliters of SFCM containing 100 μg/ml of the appropriate dead-eAP fusion protein probe and 5 μl of 1% BSA was added to the appropriate wells. After overnight incubation at 4 °C, 5 μl SFCM containing 100 μg/ml of the eAP fusion protein was added to the appropriate wells and the solutions gently mixed by pipetting. After incubation for an additional 24 h at 4 °C, the wells were washed 5 times with 300 μl pre-chilled PBST. Fifty microliters of the BluePhos AP substrate was added per well and the plate was incubated with gentle horizontal rotation at room temperature. As controls performed in parallel, the individual eAP fusion proteins and the dead-eAP fusion proteins were used as probes without dead-eAP competitor.

### Surface plasmon resonance

The affinities of FN3-MBP proteins for their bait-Fc targets were determined using a multi-cycle kinetics/affinity SPR assay with a Biacore 8K instrument (Cytiva) in the Johns Hopkins Biophysics Core laboratory. After elution from the Ni-NTA resin, 3 ml of purified FN3-MBP fusion protein was concentrated using a 10 kDa Amicon Ultra-4 centrifugation filter (Millipore Sigma). After centrifugation at 4000*g* for 20 min at 4 °C, 3 ml of storage buffer (10 mM Hepes, 150 mM NaCl, pH 7.4) was adding to the concentrated protein solution and the concentration process repeated three times to reduce the concentration of residual imidazole. The final concentrated protein solution was quantified using the Micro BCA protein assay kit (Thermo Fisher Scientific) and stored at −80 °C. Before the SPR assay, the bait-Fc protein was diluted to 10 nM with SPR running buffer HBS-EP+ buffer (Cytiva) and the FN3-MBP binding proteins were diluted to 6.25 nM, 25 nM, 100 nM, 400 nM, and 4 μM with the same running buffer.

A 10 nM solution of the bait-Fc fusion protein was injected at a flow rate of 10 μL/min for 120 s, resulting in the capture of approximately 100 RU (Response Units) of the bait protein on the surface of a protein-A Series S Sensor Chip (Cytiva). Subsequently, a single concentration of the probe was injected at a rate of 30 μL/min for 60 s. The chip was then automatically eluted with a running buffer until a stable sensorgram was obtained. Finally, the chip was regenerated using 10 mM glycine, pH 1.5 (Cytiva) to remove all captured proteins from the chip's surface, preparing it for a new cycle. Different concentrations of the binding protein were injected one-by-one in ascending order, and after completing all cycles, the data were analyzed using predefined multi-cycle kinetics/affinity parameters in the Biacore Insight Software (https://www.cytivalifesciences.com/en/us/shop/protein-analysis/spr-label-free-analysis/spr-software-and-extensions/biacore-insight-evaluation-software-p-23528). The affinities of CC4-MBP proteins for their bait-Fc proteins were determined using a five-point dose response curve and single-cycle kinetics/affinity SPR assays with a Biacore instrument and a proteinG Series S Sensor Chip (Cytiva) at Cayman Chemical, Inc.

### Immunostaining of transfected cells

HeLa or COS cells were grown in 6-well plates and, on day 1, individual wells were transfected with a single expression plasmid coding for a full-length bait protein, either a single-pass transmembrane protein or a GPI-anchored plasma membrane protein. On day 2, the transfected cells from different wells were trypsinized, mixed together, and plated on glass coverslips in the wells of a 12-well plate. On day 3, unfixed (*i.e.*, live) cells on coverslips were incubated at 4 °C for 2 h with the indicated CC4-Fc or FN3-Fc fusion proteins, either alone or as equimolar mixtures, in the form of SFCM diluted 1:50 in SFM with 1% BSA. After 2 h, the coverslips were washed three times with 0.8 mls PBS with 0.1 mM CaCl_2_, for 10 min each at 4 °C, followed by 1% paraformaldehyde (PFA) fixation at room temperature for 1.5 h, and then by three washes with 0.8 mls PBS with 0.1 mM CaCl_2_, for 10 min each. The coverslips were then incubated overnight at 4 C in Alexa488 goat anti-rabbit, Alexa594 goat anti-chicken, and Alexa647 goat anti-mouse secondary antibodies (each secondary antibody was diluted 1:400 in PBS with 7% NGS, 0.2% Triton X-100, and 0.1 mM CaCl_2_) and with DAPI. The next day, the coverslips were washed three times with 800 μl PBS with 0.1 mM CaCl_2_ and inverted onto glass slides in Fluoromount-G.

### Engineering and testing AAV with VP1-FN3 fusions

Plasmids coding for AAV2 VP1 and for VP2+VP3 were obtained from Addgene (#65724 and #65725, respectively; (67)). The VP1 plasmid was mutated to remove an extraneous NgoM IV restriction site at position 7704–7709 (in the bacteriophage F1 origin), and then various FN3 segments were cloned between the NgoM IV and Kas I sites in VP1 (replacing an mCherry insert) and corresponding to amino acids G453-R459 (453-GTTTQSR-459) within the GH2/GH3 surface loop (39, 67). Each VP1+FN3 plasmid, the VP2+VP3 plasmid, the helper plasmid pAdDeltaF6, and the AAV reporter plasmid, pAAV-CAG-SV40NLSf-GFP-3xmiR122-WPRE-HGHpA (Addgene #183775), were co-transfected into HEK293T cells to generate a concentrated AAV stock.

To test FN3-directed AAV infection, HEK293T cells were seeded on gelatin-coated coverslips in a 24-well plate (2 × 10^5^ cells/well) and transiently transfected with plasmids coding for full-length baits (0.8 μg/well) or, as a control, grown without transfection. Twenty four hours after transfection, the medium was replaced with fresh medium, and 12 h later, an aliquot of AAV, calculated to represent 2.5 × 10^7^ particles (equivalent to an MOI = 100), was added to the medium. After 24 h, an additional 500 μl of fresh medium was added in each well. Forty eight hours after AAV infection, each coverslip was PFA fixed and incubated with an FN3-mouse Fc fusion protein directed against the relevant surface protein followed by a fluorescent secondary anti-mouse antibody and processed for confocal microscopy as described under “Immunostaining of transfected cells”.

### Engineering and testing an eCIS with FN3 fusions

The PVCpnf plasmid pAWP78-PVCpnf_pvc13-A4DARPin (Addgene# 198288) and the CRE payload plasmid pBR322-Pdp1_NTD-Cre (Addgene# 198274) were purchased from Addgene. The A4DARPin coding region, which was inserted in the distal binding domain of the tail fiber gene, was replaced with an FN3 coding region using the NEBuilder HiFi Assembly System (New England BioLabs). As part of this replacement, an Apa I restriction enzyme site was inserted after the GGSGG linker so that DNA segments could be conveniently inserted or excised with Apa I and Pst I cleavage.

eCIS particles were prepared as described by (41). The modified PVC plasmid and the CRE payload plasmid were co-electroporated into EPI300 *E. coli* with kanamycin and ampicillin selection. *E. coli* cells carrying both plasmids were inoculated into 2 ml super-broth with kanamycin and ampicillin and grown overnight at 37 °C, and then 1 ml of the overnight culture was used to innoculate 200 ml of super-broth with kanamycin and ampicillin and grown for 24 h at 30 °C. The bacteria were collected by centrifugation at 4000*g* for 30 min, resuspended in 10 ml lysis buffer (25 mM Tris–HCl pH 7.5, 140 mM NaCl, 3 mM KCl, 5 mM MgCl_2_, 200 μg/ml lysozyme, 50 μg/ml DNase I, 0.5% Triton X-100, and Protease Inhibitor Cocktail), and rotated at 37 °C for 1.5 h. After centrifugation at 4000*g* for 30 min to remove cell debris, the supernatant was collected and ultracentrifuged at 120,000*g* for 2 h at 4 °C to pellet eCIS particles. The pellets were resuspended in 1 ml cold PBS and centrifuged at 12,000*g* for 15 min at 4 °C to remove debris. The supernatant was diluted with 9 ml cold PBS and ultracentrifuged a second time at 120,000*g* for 2 h at 4 °C to repellet eCIS particles. The pellets were resuspended in 500 μl cold PBS and centrifuged at 12,000*g* for 15 min at 4 °C to remove debris. The supernatant was subjected to two rounds of filtration through a 100 kDa centrifugal filter (Amicon, Millpore) at 4000*g* for 30 min 4 °C to remove low molecular weight contaminants. The protein concentration of the final eCIS particle preparation was measured with the bicinchoninic acid method (Thermo Fisher Scientific). We used freshly prepared eCIS particles as these have greater activity than eCIS particles subjected to a freeze-thaw cycle.

For cargo delivery assays, HEK293T cells were grown in 6-well plates and each well was transfected with 2 μg of loxP-stop-loxP(LSL)-nuclear localization signal-tdTomato reporter plasmid with or without 2 μg of an EPHA4 expression plasmid. Twenty four hours later, the cells were trypsinized and replated on gelatin-coated glass coverslips in the wells of a 24-well plate. An aliquot of the enriched eCIS particle preparation (corresponding to 50 μg of total protein) was added to each well in 500 μl cell culture medium. As a positive control, 1 μg of a CRE-expressing plasmid (iCre) was transfected into a control well. Thirty six hours later, the coverslips were fixed with PFA and incubated with anti-EPHA4 FN3-mouse Fc fusion protein followed by a fluorescent secondary anti-mouse antibody to visualize EPHA4 and processed for confocal microscopy as described under “Immunostaining of transfected cells”.

### Microscopy and image processing

Confocal images were captured with a Zeiss LSM700 confocal microscope using Zen Black 2012 software and processed with Fiji-ImageJ, Adobe Photoshop, and Adobe Illustrator.

### Statistical analyses

Statistical values are presented as mean ± SD. The Wilcoxon rank sum test was used to measure statistical significance.

### Materials

The sources of reagents not described above are as follows: Synthetic DNA (IDT); Alexa-Fluor conjugated secondary antibodies (Molecular Probes/Invitrogen/Thermo Fisher Scientific), AP substrate (Kirkegaard and Perry Laboratories), proteinG- and Streptavidin-coated magnetic beads (Dynal/Thermo Fisher Scientific), proteinG-coated 96-well plates (Pierce/Thermo Fisher Scientific).

## Data availability

All data for constructing binding proteins are contained in the manuscript (see Tables S2 and S3).

## Supporting information

This article contains supporting information.

## Conflict of interest

The authors declare that they have no conflicts of interest with the contents of this article.
