# Supplementary material for: Utility of protein–protein binding surfaces composed of anti-parallel alpha-helices and beta-sheets selected by phage display
Source: J Biol Chem. 2024 Apr 11;300(5):107283. doi: 10.1016/j.jbc.2024.107283 (PMC11107207; doi:10.1016/j.jbc.2024.107283)
Supplement: Supporting Table S1 [file mmc1.doc]

Supporting Information Table 1. Bait proteins. All bait proteins are of mouse origin.

Bait Amino acids

APCDD1 26-495

DKK3 1-349 (complete coding region)

EFNA1 1-186

EPHA4 1-547

FLRT1 1-544

FLRT2 1-539

FLRT3 1-526

FZD8(CRD) 1-173

LDLR 1-790

LGR4 1-542

LPHN1 1-409

PLVAP 60-452

RECK(CC1-5) 1-344
