# Supplementary material for: Utility of protein–protein binding surfaces composed of anti-parallel alpha-helices and beta-sheets selected by phage display
Source: J Biol Chem. 2024 Apr 11;300(5):107283. doi: 10.1016/j.jbc.2024.107283 (PMC11107207; doi:10.1016/j.jbc.2024.107283)
Supplement: Supporting Table S4 [file mmc4.doc]

Supporting Information Table 4. Binding proteins tested experimentally and their associated library names. The table is organized by bait and by binder scaffold type (CC4 and FN3).

CC4 scaffold

Bait binder

APCDD1 PMS33

DKK3 PMS77

FLRT1 PMS78

FLRT2 PMS19

FLRT3 PMS80

FZD8(CRD) PMS130

LGR4 PMS166

RECK(CC1-5) PMS91

FN3 scaffold

Bait binder

APCDD1 PMS267

EFNA1 PMS176

EPHA4 PMS180, PMS268, NY5

FLRT2 PMS264

FLRT3 PMS177, PMS265, NY1

LDLR PMS266

LPHN1 PMS263

PLVAP PMS181

RECK(CC1-5) PMS175, PMS262
