# Supplementary material for: Utility of protein–protein binding surfaces composed of anti-parallel alpha-helices and beta-sheets selected by phage display
Source: J Biol Chem. 2024 Apr 11;300(5):107283. doi: 10.1016/j.jbc.2024.107283 (PMC11107207; doi:10.1016/j.jbc.2024.107283)
Supplement: Supporting Information [file mmc5.doc]

Supporting Information Figure 1. (A) Left, genetic code table showing the codons generated with (A,C,G)NN. Right, genetic code table showing the codons generated with (A,C,G)N(C,G). Red, codons excluded. Black, codons included. (B) Fractional abundances of nucleotides compiled from 10^6^ NGS reads of the unselected CC4 library generated with the (C,A,G)NN strategy. A, C, and G in the first position and A, C, G, and T in the second and third positions are present at approximately equal frequency in each variable position. The corresponding amino acid sequence (in the single letter code, with “X” representing the randomized position) is shown below.

Supporting Information Figure 2. Quantitative analysis of CC4 and FN3 sequence clusters. Scatterplots for pairwise comparisons of percent amino acid sequence identity corresponding to the dendrograms in Figure 3C-E. The percent identity values are quantized, which reflects the small number of randomized codons. For each scatterplot, the sequence listed above the plot is compared to the sequences listed below the plot, i.e. all pairwise comparisons except to itself. The comparisons in (A), (B), and (C) correspond to dendrograms (C), (D), and (F), respectively. Statistical values are presented as mean ± SD. The Wilcoxon rank sum test was used to measure statistical significance.

Supporting Information Table 2. Binding protein sequences used to construct the dendrograms in Figure 3C-E. The name of each sequence (e.g. PMS262-1) is a juxtaposition of the library name (e.g. PMS262) and the rank (e.g. 1). The number of read counts following two rounds of enrichment is listed in the third column. The three parts of the table correspond to the three dendrograms in Figure 3: CC4 dendrogram (Figure 3C), FN3(beta-sheet library) dendrogram (Figure 3D), and FN3(beta-sheet + two loops library) dendrogram (Figure 3E).

Supporting Information Table 3. FN3 binding protein sequences enriched following 4 M urea washes with baits EPHA4-Fc (NY5-3 and NY5-7) and FLRT3-Fc (NY1-2, NY1-3, and NY1-4). The format is the same as for Supporting Information Table 2.
