# Supplementary material for: Utility of protein–protein binding surfaces composed of anti-parallel alpha-helices and beta-sheets selected by phage display
Source: J Biol Chem. 2024 Apr 11;300(5):107283. doi: 10.1016/j.jbc.2024.107283 (PMC11107207; doi:10.1016/j.jbc.2024.107283)
Supplement: Supporting Figure S1 [file mmc6.pdf]

A

Randomized codons: (C,A,G)NN
included (black), excluded (red)

TTT (F)	TCT (S)	TAT (Y)	TGT (C)
TTC (F)	TCC (S)	TAC (Y)	TGC (C)
TTA (L)	TCA (S)	TAA (*)	TGA (*)
TTG (L)	TCG (S)	TAG (*)	TGG (W)
CTT (L)	CCT (P)	CAT (H)	CGT (R)
CTC (L)	CCC (P)	CAC (H)	CGC (R)
CTA (L)	CCA (P)	CAA (Q)	CGA (R)
CTG (L)	CCG (P)	CAG (Q)	CGG (R)
ATT (I)	ACT (T)	AAT (N)	AGT (S)
ATC (I)	ACC (T)	AAC (N)	AGC (S)
ATA (I)	ACA (T)	AAA (K)	AGA (R)
ATG (M)	ACG (T)	AAG (K)	AGG (R)
GTT (V)	GCT (A)	GAT (D)	GGT (G)
GTC (V)	GCC (A)	GAC (D)	GGC (G)
GTA (V)	GCA (A)	GAA (E)	GGA (G)
GTG (V)	GCG (A)	GAG (E)	GGG (G)

Randomized codons: (C,A,G)N(C,G)
included (black), excluded (red)

TTT (F)	TCT (S)	TAT (Y)	TGT (C)
TTC (F)	TCC (S)	TAC (Y)	TGC (C)
TTA (L)	TCA (S)	TAA (*)	TGA (*)
TTG (L)	TCG (S)	TAG (*)	TGG (W)
CTT (L)	CCT (P)	CAT (H)	CGT (R)
CTC (L)	CCC (P)	CAC (H)	CGC (R)
CTA (L)	CCA (P)	CAA (Q)	CGA (R)
CTG (L)	CCG (P)	CAG (Q)	CGG (R)
ATT (I)	ACT (T)	AAT (N)	AGT (S)
ATC (I)	ACC (T)	AAC (N)	AGC (S)
ATA (I)	ACA (T)	AAA (K)	AGA (R)
ATG (M)	ACG (T)	AAG (K)	AGG (R)
GTT (V)	GCT (A)	GAT (D)	GGT (G)
GTC (V)	GCC (A)	GAC (D)	GGC (G)
GTA (V)	GCA (A)	GAA (E)	GGA (G)
GTG (V)	GCG (A)	GAG (E)	GGG (G)

B

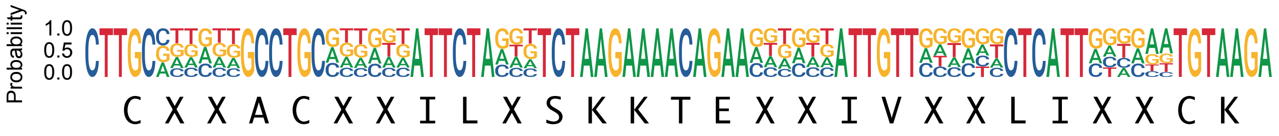

Zhu et al., SI Figure 1
