# Supplementary figures and images for: Utility of protein–protein binding surfaces composed of anti-parallel alpha-helices and beta-sheets selected by phage display
Source: J Biol Chem. 2024 Apr 11;300(5):107283. doi: 10.1016/j.jbc.2024.107283 (PMC11107207; doi:10.1016/j.jbc.2024.107283)

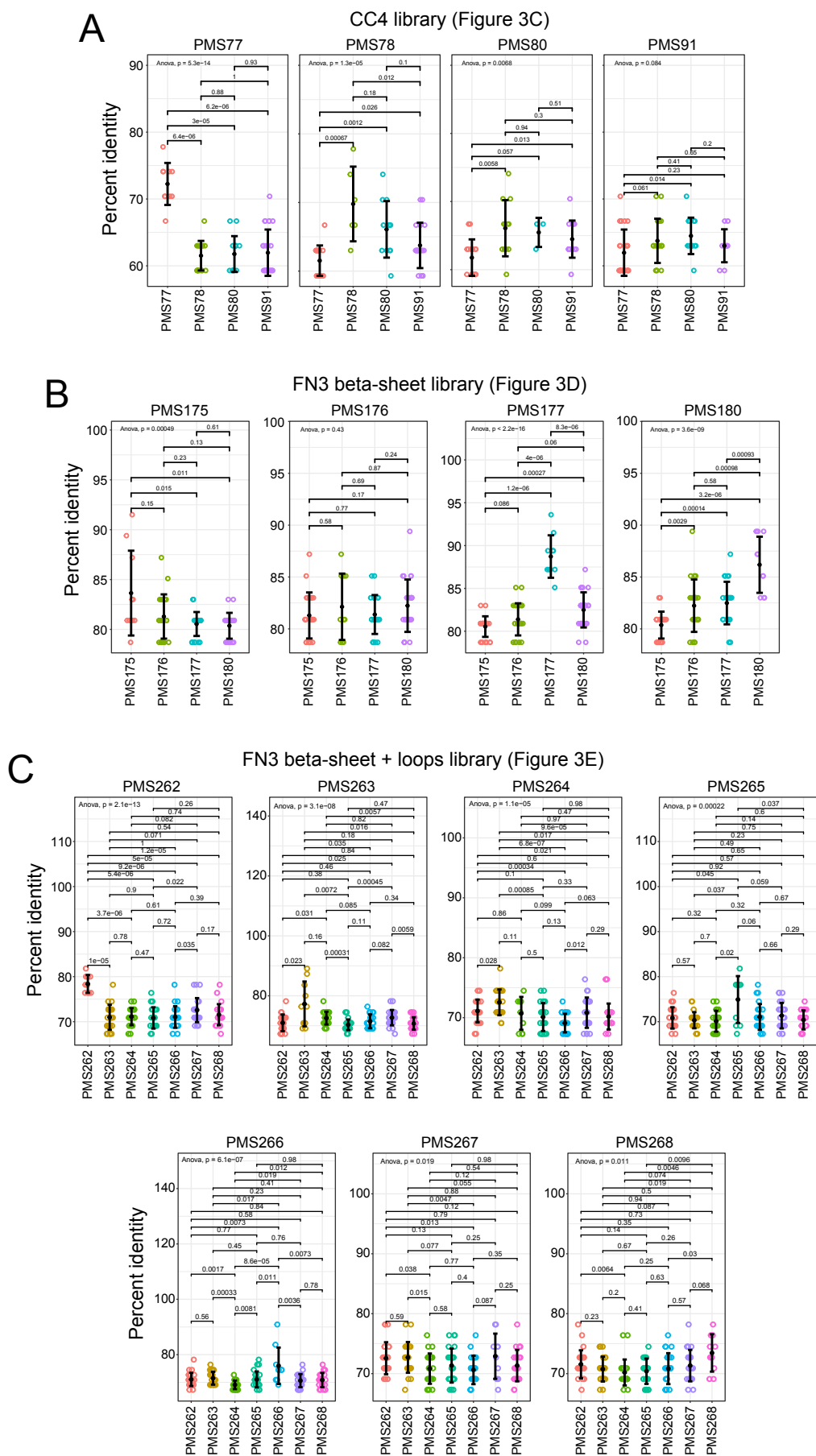

Supplement: Supporting Figure S2 [file mmc7.pdf]
